# A2780 human ovarian cancer cells with acquired paclitaxel resistance display cancer stem cell properties

**DOI:** 10.3892/ol.2013.1568

**Published:** 2013-09-06

**Authors:** XIAOFENG HAN, FANGFANG DU, LI JIANG, YIFEI ZHU, ZHEN CHEN, YANJUN LIU, TINGTING HONG, TENG WANG, YONG MAO, XIAOHONG WU, IAIN C. BRUCE, JIAN JIN, XIN MA, DONG HUA

**Affiliations:** 1 Schools of Medicine, Jiangnan University, Wuxi, Jiangsu 214122, P.R. China; 2 Pharmaceutical Sciences, Jiangnan University, Wuxi, Jiangsu 214122, P.R. China; 3 Affiliated Hospital, Jiangnan University, Wuxi, Jiangsu 214122, P.R. China

**Keywords:** ovarian cancer, cancer stem cells, aldehyde dehydrogenase 1, paclitaxel, chemoresistance

## Abstract

The use of chemotherapy to treat cancer is effective, but chemoresistance reduces this efficacy. Chemotherapy resistance involves several mechanisms, including the cancer stem cell (CSC) concept. The aim of the present study was to assess whether paclitaxel-resistant epithelial ovarian carcinoma is capable of generating cells with CSC-like properties. Using the paclitaxel-resistant A2780/PTX cell line, it was demonstrated that high aldehyde dehydrogenase 1 (ALDH1) activity identifies CSCs from diverse sources. Furthermore, the A2780/PTX cells had a strong ability to form colonies in soft agar assays. Notably, it was demonstrated that the inhibition of the PI3K signaling pathway abolished colony formation. These data suggest that there is a link between paclitaxel resistance and CSC enrichment. It is possible that therapeutic benefits, such as the restoration of chemosensitivity or the suppression of tumorigenicity, may be enabled by gaining further insights into the mechanisms underlying chemoresistance and the generation of CSCs.

## Introduction

Ovarian cancer is the fifth most common type of cancer in females and the leading cause of mortality among gynecological malignancies, with epithelial carcinoma being the most frequent variety (1,2). Despite advances in surgery and chemotherapy, the survival rate of patients with epithelial ovarian cancer remains extremely low (3). Furthermore, the majority of patients relapse and even become drug-resistant (4). Increasing evidence indicates that the initiation, progression, recurrence and metastasis of cancer are associated with the behavior of cancer stem cells (CSCs) (5). CSCs have been defined as a small subpopulation of cells within the tumor bulk mass that possess the capacity to self-renew and give rise to all heterogeneous cancer cell lineages that comprise the tumor of origin. CSCs are identified and isolated by the expression of distinctive markers for enrichment (6).

Aldehyde dehydrogenase 1 (ALDH1) is a candidate marker for cancer cells with stem cell-like properties (7). ALDH1 is from a family of intracellular enzymes that participate in cellular detoxification, differentiation and drug resistance through the oxidation of intracellular aldehydes (8). It has been demonstrated that mouse and human hematopoietic and neural stem and progenitor cells have high ALDH1 activity (9). High ALDH1 activity associated with poor clinical outcome has been reported in breast cancer cells (10), ovarian cancer cells (11) and glioblastomas (12). Therefore, the use of ALDH1 activity as a purification strategy allows the non-toxic and efficient isolation of human stem-like cells, based on a developmentally conserved stem/progenitor cell function (13). The PI3K/Akt signaling pathway is rapidly emerging as a signaling network that is important for CSC survival (14). Therefore, the therapeutic targeting of the PI3K/Akt axis by small molecule inhibitors is being pursued as an option for the innovative treatment of several types of cancer.

The present study assessed the expression of ALDH1 in human ovarian cancer cells: Wild-type A2780/WT cells and the related paclitaxel-resistant A2780/PTX cell line. The colony formation assay was used to characterize differences accompanied by ALDH1 expression. It was identified that PI3K inhibition abolished the colony formation by the A2780/PTX cells. The findings suggest that cells with acquired paclitaxel resistance display CSC properties, with enhanced malignant and tumor-forming properties.

## Materials and methods

### Cells and reagents

The A2780/WT and A2780/PTX cell lines were obtained from KeyGen Biotech Co., Ltd. (Nanjing, China) and maintained in RPMI-1640 supplemented with 10% fetal bovine serum and penicillin-streptomycin (100 U/ml penicillin and 100 μg/ml streptomycin; Invitrogen Life Technologies, Carlsbad, CA, USA) at 37°C in a humidified atmosphere of 5% CO_2_. The paclitaxel was obtained from Qilu Pharmaceutical Co., Ltd. (Jinan, China), the LY294002 was from Selleckchem (Burlington, NC, USA) and the agarose was from Sigma-Aldrich (St. Louis, MO, USA).

### Analysis of ALDH activity

To assess ALDH activity in the two cell lines, the Aldefluor™ kit (StemCell Technologies, Vancouver, BC, Canada) was used according to the manufacturer’s instructions. In brief, cells were suspended (1×10^6^ cells/ml) in the Aldefluor assay buffer containing the ALDH substrate (Bodipy^®^-aminoacetaldehyde) and incubated for 45 min at 37°C to allow substrate conversion. As a negative control for all experiments, the cells were suspended in buffer containing the Aldefluor substrate in the presence of diethylaminobenzaldehyde (DEAB), a specific ALDH enzyme inhibitor. The cells were analyzed using a FACSCalibur flow cytometer (BD Biosciences, Franklin Lakes, NJ, USA) and the data were analyzed using FlowJo software, version 7.6.1 (Treestar Inc., Ashland, OR, USA).

### In vitro soft agar for colony formation assay

In preparation for the assay, 1% agarose in normal growth medium was coated onto 12-well plates and allowed to cool for 1 h at room temperature. Suspensions of each cell type (2.0×10^3^ cells/well) were prepared using 0.4% agarose in normal growth medium and plated on top of the 1% agarose base layer. Subsequently, normal growth medium was applied on top of the cell layer and changed every three days for two weeks. Images were captured with a Panasonic Lumix DMC-FH3 camera (Panasonic, Osaka, Japan). If needed, the cells were pretreated with LY294002 (10 μM, two weeks).

### Involvement of the PI3K signaling pathway

To assess the involvement of the PI3K signaling pathway in colony formation, LY294002 (10 μM), a specific PI3K inhibitor, was applied to the A2780/PTX cells. DMSO (0.1%) was used as a control.

### Statistical analysis

Results are presented as the mean ± standard deviation. Student’s t-test was used to determine the statistical significance of the differences. P<0.05 indicated a statistically significant difference.

## Results

### Acquisition of paclitaxel resistance in the A2780/PTX cells induces the CSC phenotype

**Figure 1:**
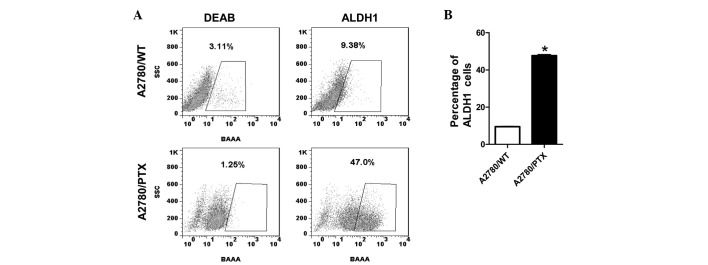
FACS analysis of ALDH-positive cells in A2780/WT and A2780/PTX cell lines. (A) Representative plots and (B) summary data showing the expression of ALDH1 in the A2780/WT and A2780/PTX cells. DEAB served as a negative control. ^*^P<0.05, compared with A2780/WT cells. FACS, fluorescence-activated cell sorting; ALDH, aldehyde dehyrogenase; DEAB, diethylaminobenzaldehyde.

To determine the effect of the acquisition of paclitaxel resistance on the CSC phenotype in the A2780 cells, the proportion of ALDH1-positive A2780/PTX cells and A2780/WT cells was measured by fluorescence-activated cell sorting analysis. The percentage of ALDH1-positive A2780/PTX cells was significantly greater than that of wild-type cells. These results suggested that the acquisition of paclitaxel resistance increased the proportion of CSCs expressing ALDH1 (Fig. 1).

### Acquisition of paclitaxel resistance in A2780/PTX cells enhances colony formation

**Figure 2:**
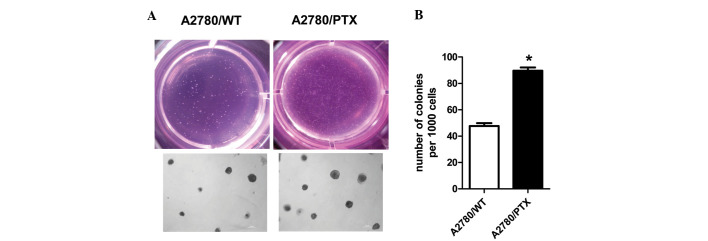
A2780/PTX cells show enhanced colony formation. (A) Representative images and (B) summary data showing colony formation. Cells were plated in soft agar in 12-well plates and images of tumor spheres were captured after 14 days (upper panels in A). Tumor spheres formed from 2,000 A2780/WT cells and A2780/PTX cells (lower panels in A). ^*^P<0.05, compared with A2780/WT cells.

A characteristic of stem cells is their ability to form clones in soft agar (15). In order to determine whether the cancer cells form such colonies, 2,000 A2780/WT cells and A2780/PTX cells were seeded into soft agar growth medium in 12-well plates and cultured for two weeks. The two cell types formed colonies, but the A2780/PTX cells were more tumorigenic than the A2780/WT cells (Fig. 2).

### Inhibition of PI3K activity blocks colony formation in A2780/PTX cells

**Figure 3:**
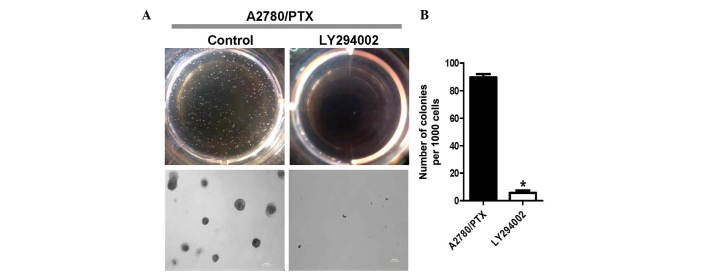
Inhibition of PI3K activity blocks colony formation in A2780/PTX cells. (A) Representative images and (B) summary data showing the effect of the PI3K inhibitor, LY294002 (10 μM). Images of the cells were captured after 14 days (upper panels in A). Tumor spheres formed from 2,000 A2780/PTX cells, with and without LY294002 treatment (lower panels in A). Control indicates DMSO (0.1%). ^*^P<0.05, compared with A2780/PTX cells without LY294002. DMSO, dimethyl sulfoxide.

To assess the involvement of the PI3K signaling pathway, the PI3K inhibitor (LY294002; 10 μM) was applied to the A2780/PTX cells. This inhibitor blocked the colony formation by the A2780/PTX cells in soft agar (Fig. 3).

## Discussion

First-line chemotherapy often leads to encouraging responses in cancer (16), however, in the course of the treatment, resistance frequently occurs and ultimately limits life expectancy (17). The concept of the CSC is one explanation for chemoresistance (18). CSCs isolated from human cancers, including ovarian cancer (2), glioblastoma (19) and breast cancer (20), have reportedly increased resistance to conventional therapies. The development of more effective therapies may require targeting this important CSC population. The success of this new approach is dependent on the identification and characterization of CSCs. Recently, ALDH1 activity has been used successfully as a marker to isolate stem cells from diverse sources (21).

The present study used wild-type (A2780/WT) and paclitaxel-resistant (A2780/PTX) human epithelial ovarian cancer cells to investigate the expression of ALDH1 and the associated capacity for colony formation. The A2780/PTX cells were shown to display high ALDH1 activity, consistent with other studies showing that a high percentage of ALDH1-positive tumor cells is significantly associated with a poor clinical outcome in serous ovarian cancer (22,23). Subsequently, colony formation by the A2780/PTX cells was examined. As predicted, the findings demonstrated that the A2780/PTX cells displayed enhanced colony formation in the soft agar assay. This indicated that the A2780/PTX cells were more tumorigenic than the A2780/WT cells.

Since the PI3K/AKT axis is frequently activated in human cancer (24), the present study explored whether these pathways are involved in the regulation of tumorigenicity. The PI3K inhibitor, LY294002, abolished colony formation when added to the A2780/PTX cells. Although further studies are required to elucidate the mechanism of PI3K-mediated colony formation, the use of PI3K inhibitors to reduce tumorigenicity may be a useful approach to improve the efficacy of chemotherapy.

In conclusion, the present study showed that the selection and isolation of stem-like ovarian cancer cells, on the basis of ALDH1 activity, revealed enhanced colony formation. Targeting the cells with the PI3K inhibitor, LY294002, abolished this capacity. We believe that these findings are of importance and support clinical trials of LY294002 in ovarian cancer, either alone or in combination with established therapies.
