# A2780 human ovarian cancer cells with acquired paclitaxel resistance display cancer stem cell properties

**DOI:** 10.3892/ol.2022.13526

**Published:** 2022-09-26

**Authors:** Xiaofeng Han, Fangfang Du, Li Jiang, Yifei Zhu, Zhen Chen, Yanjun Liu, Tingting Hong, Teng Wang, Yong Mao, Xiaohong Wu, Iain C. Bruce, Jian Jin, Xin Ma, Dong Hua

Oncol Lett 6: 1295–1298, 2013; DOI: 10.3892/ol.2013.1568

Subsequently to the publication of this paper, an interested reader drew to the authors’ attention that, in Fig. 2A and 3A on p. 1296, there were a pair of apparently overlapping data panels (specifically, the data representing the tumor spheres for the A2780/PTX cells in Fig. 2A, and the tumor spheres for the control A2780/PTX cells in Fig. 3A). The authors have re-examined their data, and realized that the image shown correctly for Fig. 2A was inadvertently and erroneously included in Fig. 3A.

The authors have reassembled Fig. 3, and the corrected version of this figure, now showing the correct data for the tumor spheres for the control A2780/PTX cells in Fig. 3A, is shown opposite. Note that this error did not have a major impact on either the results or the conclusions reported in this paper. The authors regret that this error went uncorrected before the paper was published, and are grateful to the Editor of *Oncology Letters* for allowing them to publish this Corrigendum. All the named authors agree to the publication of this Corrigendum, and apologize to the readership for any inconvenience caused.

## Figures and Tables

**Figure 3.**
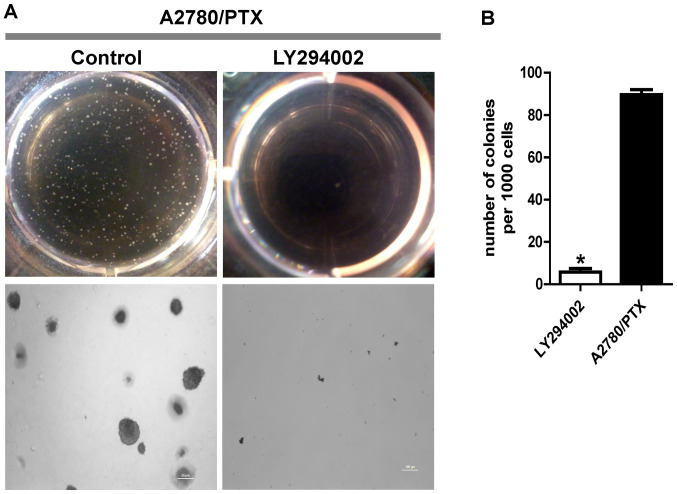
Inhibition of PI3K activity blocks colony formation in A2780/PTX cells. (A) Representative images and (B) summary data showing the effect of the PI3K inhibitor, LY294002 (10 μM). Images of the cells were captured after 14 days (upper panels in A). Tumor spheres formed from 2,000 A2780/PTX cells, with and without LY294002 treatment (lower panels in A). Control indicates DMSO (0.1%). *P<0.05, compared with A2780/PTX cells without LY294002. DMSO, dimethyl sulfoxide.

